# Heritability and Environmental Correlation of Phase Angle with Anthropometric Measurements: A Twin Study

**DOI:** 10.3390/ijerph17217810

**Published:** 2020-10-26

**Authors:** Daisuke Matsumoto, Fujio Inui, Chika Honda, Rie Tomizawa, Mikio Watanabe, Karri Silventoinen, Norio Sakai

**Affiliations:** 1 Department of Physical Therapy, Faculty of Health Sciences, Kio University, 4-2-2 Umaminaka, Koryo-cho, Kitakatsuragi-gun, Nara 635-0832, Japan; 2 Health Promotion Center, Kio University, 4-2-2 Umaminaka, Koryo-cho, Kitakatsuragi-gun, Nara 635-0832, Japan; f.inui@kio.ac.jp; 3 Center for Twin Research, Osaka University Graduate School of Medicine, Suita, Osaka 565-0871, Japan; honda-ch@sahs.med.osaka-u.ac.jp (C.H.); r-tomizawa@sahs.med.osaka-u.ac.jp (R.T.); nabe@sahs.med.osaka-u.ac.jp (M.W.); karri.silventoinen@helsinki.fi (K.S.); norio@sahs.med.osaka-u.ac.jp (N.S.); 4 Department of Nursing, Faculty of Health Sciences, Kio University, 4-2-2 Umaminaka, Koryo-cho, Kitakatsuragi-gun, Nara 635-0832, Japan; 5 Faculty of Nursing, Shiga University of Medical Science, Seta Tsukinowa-cho, Otsu, Shiga 520-2192, Japan; 6 Department of Clinical Laboratory and Biomedical Sciences, Division of Health Sciences, Osaka University Graduate School of Medicine, Suita, Osaka 565-0871, Japan; 7 Department of Social Research, Faculty of Social Sciences, University of Helsinki, P.O. Box 18, 00014 Helsinki, Finland; 8 Child Healthcare and Genetic Science Laboratory, Division of Health Sciences, Osaka University Graduate School of Medicine, Suita, Osaka 565-0871, Japan

**Keywords:** phase angle, heritability, twin study, anthropometric measurements

## Abstract

Bioelectrical impedance analysis (BIA)-derived phase angle (PhA) is a valuable parameter to assess physical health. However, the genetic and environmental aspects of PhA are not yet well understood. The present study aimed to estimate the heritability of PhA and investigate the relationships between PhA and anthropometric measurements. PhA and skeletal muscle mass index (SMI) were examined using multi-frequency BIA in 168 Japanese twin volunteers (54 males and 114 females; mean age = 61.0 ± 16.5 years). We estimated the narrow-sense heritability of these parameters and the genetic and environmental relationships between them using a genetic twin modeling. For the PhA, 51% (95% confidence interval: 0.33, 0.64) of the variance was explained by additive genetic effects, and 49% (95% confidence interval: 0.36, 0.67) was explained by unique environmental effects. The heritability of PhA was lower than the height, body weight, and body mass index. PhA shared almost no genetic variation with anthropometric measurements and SMI but shared an environmental variation (14%) with SMI. These findings suggest that the genes affecting PhA are different than those affecting anthropometric measurements and SMI. The correlation between PhA and SMI is caused by common environmental factors.

## 1. Introduction

Bioelectrical impedance analysis (BIA) has been widely used as a non-invasive, inexpensive, and quick technique to estimate body composition by sending a weak electric current [1,2]. BIA works mainly through the measuring of body resistance and reactance to alternate electrical current. Resistance depends on the fluid and electrolyte content of the body. Cell membranes produce reactance by storing parts of the charge as a capacitor. This storage of the current creates a phase shift that can be seen as the ratio of resistance and reactance and is presented geometrically as the phase angle (PhA) [3]. PhA is one of the parameters of BIA that is related to cell size or integrity (structure) of the cell membrane [3], and it may be a suitable marker for nutritional assessment [4,5]. A systematic review [6] found that 42 of 48 selected studies showed a correlation between low PhA and mortality. Thus, PhA seems to be a good predictor of mortality in many clinical situations, especially kidney disease and cancer, and can be used for screening individuals prone to these diseases. Recently, researchers have proposed that PhA is a good indicator of sarcopenia [7], falling [8], frailty [9], incident disability [10], and quality of life [11].

In addition, age-related changes in PhA have been reported previously in the context of whole-body measures of body composition [12]. PhA is related to muscle mass and strength in elderly subjects [13], and 12 weeks of resistance training in untrained older women can improve PhA. Furthermore, changes in muscle quality are positively correlated with changes in PhA [14]. According to the European Working Group on Sarcopenia in Older People (EWGSOP2), muscle quality can be assessed by a BIA-derived PhA measurement [15]. In the future, assessments of muscle quality are expected to guide treatment choices and monitor response to treatment [16].

Anthropometric measurements such as height, body weight, body mass index (BMI), and muscle mass phenotypes are influenced by genetic factors [17,18,19]. The heritability of BMI (0.51–0.77) is found to be largely similar in different populations and time periods despite large differences in mean BMI and variances of BMI [19]. Moreover, body fat mass and free-fat mass were also high but less heritable than height, body weight, and BMI [20,21]. Despite the previously mentioned prognostic potentials of PhA, there are no studies assessing the heritability of PhA. We hypothesized that the heritability of PhA would be lower than height, body weight, BMI, and muscle mass and have less genetic influence. Therefore, using structural equation modelling (SEM), the present study aimed to estimate the heritability of PhA. In addition, we examined whether PhA share the same genetic or environmental variation with anthropometric measurements by Cholesky decomposition.

## 2. Materials and Methods

### 2.1. Participants

The twin volunteers were recruited from the registry established by the Center for Twin Research, Osaka University, between June 2018 and December 2019. Eligibility criteria of the present study were the following: (1) Aged 20 years or older, (2) same-sex twin pairs, and (3) no dementia. We examined a total of 168 participants (54 males and 114 females): 75 monozygotic (MZ) twin pairs (*n* = 150) and nine same-sex dizygotic (DZ) twin pairs (*n* = 18).

The study protocol was approved by the ethics committee of the Osaka University (696-5 and 10209-15). All study participants provided informed consent, and participant anonymity was preserved. The study was conducted according to the provisions of the Declaration of Helsinki.

### 2.2. Measurements

Height was measured with a stadiometer to the nearest 0.1 cm while standing without shoes. A body composition analyzer (MC-780A; TANITA Co., Ltd., Tokyo, Japan) was used to determine body weight and bioelectrical impedance. Participants were instructed to stand bare feet on the metal footplate of the analyzer, holding the metallic grip electrodes with arms straight and pointed downwards in a neutral standing position. We performed the examination in the morning after 12 h of fasting and sufficient rest to optimize the results of the BIA analysis. In addition, the skeletal muscle mass index (SMI) was determined as the appendicular skeletal muscle mass (kg) divided by height squared [16,22]. Resistance (R) and reactance (Xc) measured at 50 kHz were used for calculation of the PhA with the following equation [8]:

PhA (degrees) = −arc tangent (Xc/R) × (180/π).

### 2.3. Statistical Analysis

To evaluate the relative importance of genetic and environmental factors for the measured phenotype, we used SEM. Individual differences in a trait can be decomposed into genetic and environmental sources of variance [23]. The sources of genetic and environmental variation considered in behavioral genetics are as follows: Additive genetic factors (A); dominance genetic factors (D); environmental factors shared by both twins (family member) (C); and environmental factors unique to each twin individual and also measurement error (E). SEM represents a unified platform for path analysis and variance components of models and is the current method that is used to analyze twin data [24]. The heritability, which is calculated by SEM, was called the narrow-sense heritability. Because (C) and (D) components cannot be estimated simultaneously in the classical twin model with twins reared together, either the ACE or ADE model is fitted [25]. In this study, we illustrate the ACE model as an example. In the model, the following possible parameter combinations are considered: ACE, AE, CE, and E.

**Figure 1:**
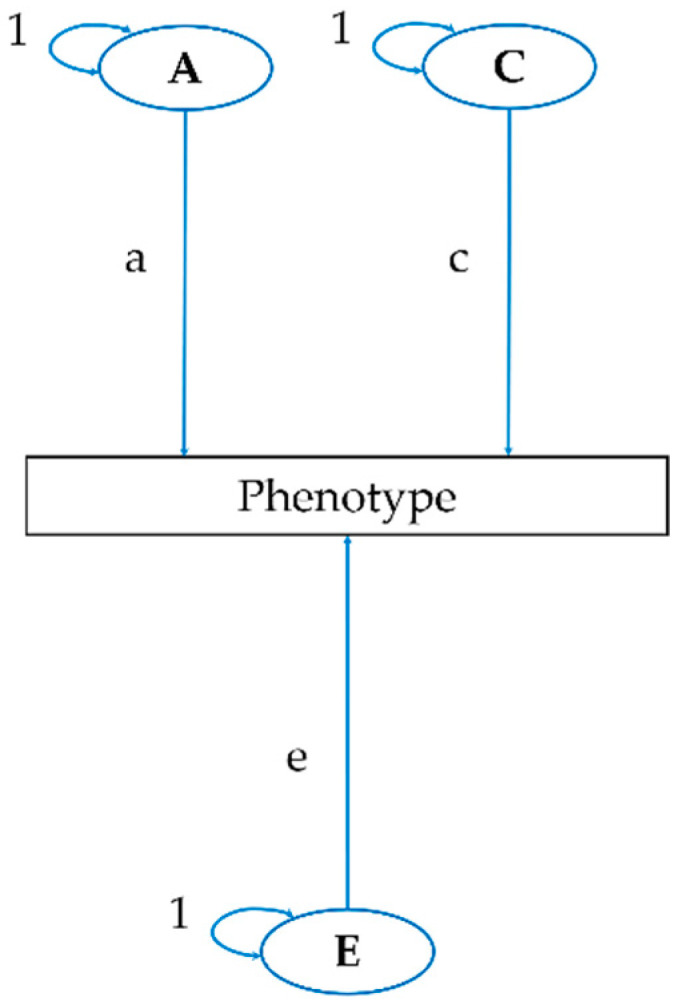
Path diagram for the univariate ACE model. (A) Additive genetic factor; (a) the path from a genetic factor; (C) shared environmental factor; (c) the path from a shared environmental factor; (E) non-shared environmental factor; (e) the path from a non-shared environmental factor.

A full model (ACE model), including all latent variables, was examined against nested sub-models (AE, CE, and E) with the reduced numbers of parameters to find the most parsimonious model. The fittings of the alternative models were compared with the difference in −2 log likelihood, which is asymptotically distributed as χ^2^ with degrees of freedom equal to the difference in the number of parameters. The fittings of the different models were also analyzed according to Akaike’s information criterion [26], in which a smaller value indicates a better model. The estimates with 95% confidence intervals (CI) were obtained from the theoretically most acceptable and most parsimonious model, respectively. A path diagram of the univariate ACE model is shown in Figure 1, and the model represents the equation, which follows.

P = aA + cC + eE

V_P_ = a^2^ + c^2^ + e^2^

The variables represent the following: P = phenotype; V_P_ = total variance of the phenotype; a = the path from a genetic factor; c = the path from a shared environmental factor; and e = the path from a non-shared environmental factor.

**Figure 2:**
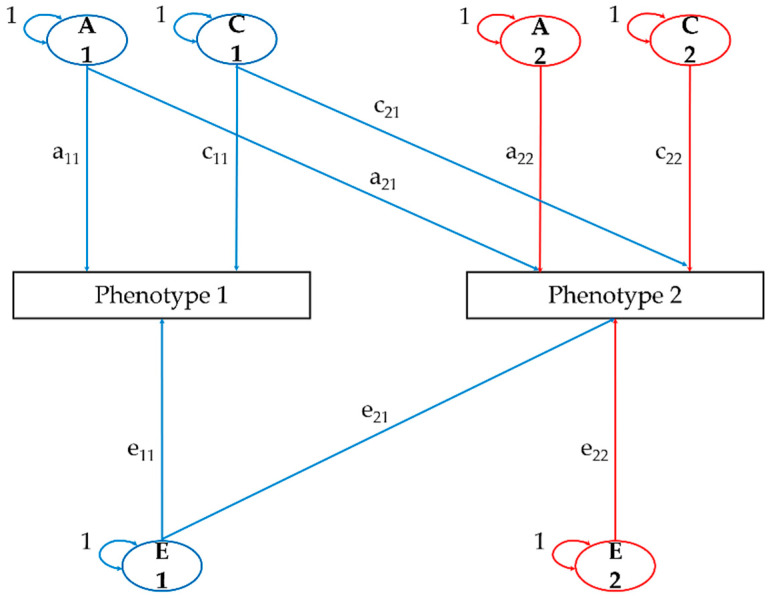
Path diagram for the bivariate Cholesky decomposition model. Subscripts with paths indicate a path direction and an origin (e.g., for a_21_, 2 is the path direction phenotype 2, and 1 is the origin). (A) Additive genetic factor; (a) the path from a genetic factor; (C) shared environmental factor; (c) the path from a shared environmental factor; (E) non-shared environmental factor; (e) the path from a non-shared environmental factor.

Furthermore, we used Cholesky decomposition to evaluate the relationship between phenotypes [27]. In the Cholesky decomposition, the first factor influences all the variables, the second factor influences all of them except for the first one, and so on. A path diagram of the bivariate Cholesky decomposition model is shown in Figure 2. The model represents the equation, which follows.

P1 = (a_11_A_1_ + c_11_C_1_ + e_11_E_1_)

P2 = (a_21_A_1_ + a_22_A_2_ + c_21_C_1_ + c_22_C_2_ + e_21_E_1_ + e_22_E_2_)

V_P_ 1 = a_11_^2^ + c_11_^2^ + e_11_^2^

V_P_ 2 = a_21_^2^ + a_21_^2^ + c_21_^2^ + c_22_^2^ + e_21_^2^ + e_22_^2^

The variables represent the following: P1 = phenotype 1; V_P_ 1 = total variance of the phenotype 1; P2 = phenotype 2; V_P_ 2 = total variance of the phenotype 2.

Student’s t test was used to compare all variables between males and females. Pearson’s correlations were used to determine the correlations among all parameters in MZ and DZ twins. Statistical analysis was performed using IBM SPSS Statistics for Windows (version 26.0J, IBM Japan Corp., Tokyo, Japan) and twin SEM was implemented in R (version 3.6.3 for Windows, R Development Core Team, Vienna, Austria) Package OpenMx (version 2.17, OpenMx Development Core Team, Virginia, USA), adjusted for age and sex [28].

## 3. Results

Table 1 shows the comparison of height, body weight, BMI, SMI, and PhA by sex. No significant difference was observed for age. All parameters in males were higher than those in females (*p* < 0.001). Pearson correlations between anthropometric measurements and PhA in the MZ and DZ groups are presented in Table 2. Of all measurements, only SMI was most strongly correlated with PhA in both the MZ group (*r* = 0.390) and the DZ group (*r* = 0.605). We found that the relationships between SMI and PhA were stronger in the DZ group than the MZ group. No significant correlation was observed between PhA and height or body weight.

The results of univariate ACE and ADE model fitting and estimated values are shown in Table 3 and Table S1. In the ACE and ADE model (a full model), these models were not fitting, and we cannot estimate values in all phenotypes. In ACE model, the AE model was the best-fitting model because it had lower −2 log likelihood and Akaike’s information criterion than CE model. In addition, the *p*-value suggested that a significant difference was not present between the ACE model and the AE model. Therefore, shared environmental (C) effects had no impact on the total variance of all the phenotypes that were considered. The narrow-sense heritability was estimated using the univariate AE models for all phenotypes. The heritability estimate for height was 0.932 (95% CI: 0.908–0.950), for body weight was 0.758 (95% CI: 0.682–0.817), for BMI was 0.718 (95% CI: 0.630–0.786), and for SMI was 0.513 (95% CI: 0.457–0.625). For the PhA, 51% (95% CI: 0.341–0.642) of the variance was explained by additive genetic effects, and 49% (95% CI: 0.358–0.659) was explained by unique environmental (including measurement error) effects (Table 3).

**Figure 3:**
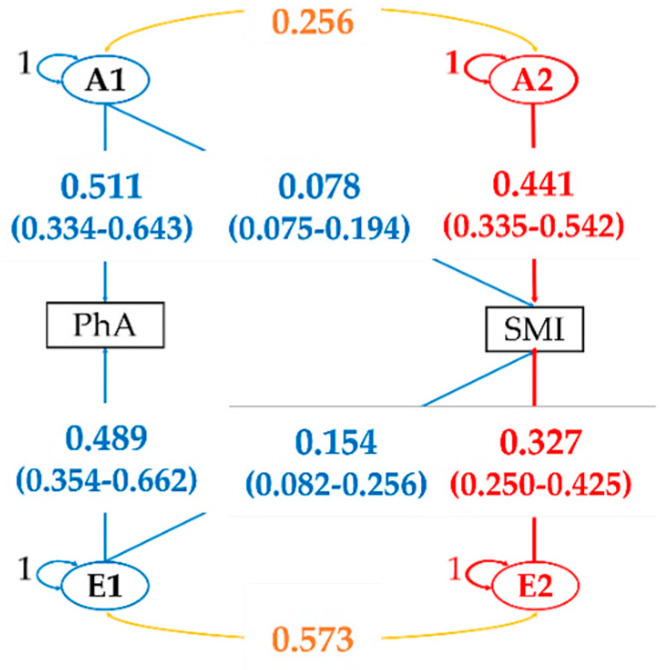
Cholesky decomposition path diagram between phase angle and skeletal muscle mass index for the AE model. Standardized path coefficients are presented. Numbers in parentheses are 95% confidence intervals of estimates. The number between (A1) and (A2) and the number between (E1) and (E2) are correlation coefficients. (A1–2) Additive genetic factor; (E1–2) Non-shared environmental factor; PhA: Phase angle; SMI: Skeletal muscle mass index.

**Figure 4:**
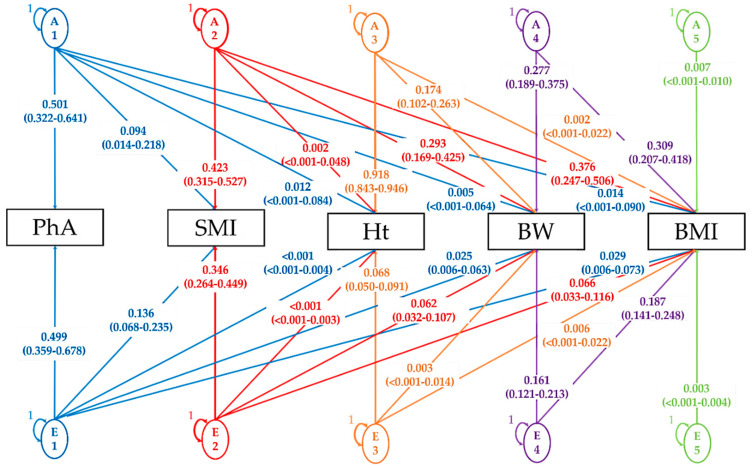
Cholesky decomposition path diagram among anthropometric measurements and phase angle for the AE model. Standardized path coefficients are presented. Numbers in parentheses are 95% confidence intervals of estimates. (A1–A5) additive genetic factor; (E1–5) non-shared environmental factor; PhA: Phase angle; SMI: Skeletal muscle mass index; Ht: Height; BW: Body weight; BMI: Body mass index.

Next, we applied the AE model to perform Cholesky decomposition. The standardized path coefficients are shown in Figure 3 and Figure 4. In the bivariate analysis between PhA and SMI, we found that the non-shared environmental factor e_21_ (15%) contributed a higher proportion of the total variance of VP2 than additive genetic factor a_21_ (8%) (Figure 3). Figure 4 shows the Cholesky decomposition path diagram among all phenotypes for the AE Model. Although A1 (common additive genetic factor) explained 50% of VP1 for PhA, it explained less than 10% of variance for the other phenotypes. For E1 explained by unique environmental factors for PhA, e_21_ (14%), which was common with SMI, contributed the highest proportion of variance.

## 4. Discussion

In our study, we aimed to estimate the heritability of PhA and to investigate the genetic and environmental aspects of the relationships between anthropometric measurements and PhA. The distribution of PhA and the other measurements in this study was relatively normal for Asians. PhA usually ranges between 5–7° in healthy adults and is usually lower in women than men [29,30]. Our results confirmed that height (0.932), body weight (0.758) and BMI (0.718) were highly heritable, similar to previous studies that reported that, for example, the heritability of BMI was 0.51–0.81 for 20–79 years old [20,21].

Our main results for PhA showed that additive genetic effects explained 51% of the variance, whereas unique environmental effects explained the rest of the variance. The heritability of PhA was lower than that of anthropometric measurements such as BMI. It is the first study to clarify the heritability of BIA-derived PhA. This finding could help more in the accurate interpretation of PhA. These results indicate that PhA is more strongly affected by environmental effects, that is, it is more modifiable than BMI.

Cholesky decomposition showed that PhA shared little genetic and environmental effects with height, body weight, or BMI. In fact, PhA is an independent parameter. Environmental factors for PhA contribute to more variance in SMI than genetic factors. That is, the phenotypic relationship between PhA and SMI was mediated in part by common environmental influences. The unique environment includes measurement error in the classical twin design [23]. However, this correlation was unexplainable by only measurement error. The cause of the phenotypic relationship between PhA and SMI may be explained by the relationship to sarcopenia [31,32]. For example, resistance training (RT) has a common environmental effect on both PhA and SMI. Although the physiological mechanisms by which RT improves PhA are not fully understood, the PhA change due to RT may be attributed to some known factors [33,34,35]. PhA can be affected by changes in the capacitive behavior of the tissues, cell size and mass, cell membrane permeability, or intracellular composition [36]. RT-induced changes seem to counteract age-related decreases in PhA [33]. However, genetic factors may explain the individual variation in the response to this intervention [37]. An intervention needs to be adjusted to the patient’s response as indicated by parameters such as PhA and SMI [38].

This study has some limitations. First, these findings were derived from a cross-sectional design. Second, the sample size of the study was small, particularly for the DZ group. Third, PhA may be affected by physical activity [39] and nutritional status [3,4,5]. Finally, because our participants were Japanese, application to other races, ethnicities, and residents of other countries is difficult. Future studies with a longitudinal design, larger number of participants, more potential confounding factors, and international collaborations are needed to confirm our results.

## 5. Conclusions

In conclusion, this study provided new insights into the genetic and environmental effects on PhA. Our findings suggest that PhA could be a practical and independent parameter of genetic determinants of physical health. The correlation between PhA and SMI was explained more strongly by environmental factors than genetic factors.

## Figures and Tables

**Table 1:** A comparison of anthropometric measurements and PhA in males and females.

Variable	Total (*n* = 168)		Male (*n* = 54)		Female (*n* = 114)		
	Mean	SD	Mean	SD	Mean	SD	*p*-Value
Age (years)	61.0	16.5	60.7	20.9	61.1	14.0	0.903
Height (cm)	157.8	8.23	165.9	6.30	153.9	5.95	<0.001
Body Weight (kg)	53.8	10.3	63.0	9.57	49.5	7.34	<0.001
BMI (kg/m^2^)	21.5	3.19	22.9	3.32	20.9	2.93	<0.001
SMI (kg/m^2^)	6.79	1.12	7.95	0.93	6.22	0.67	<0.001
PhA (°)	5.46	0.88	5.94	0.92	5.24	0.76	<0.001

Note: SD: Standard deviation; BMI: Body mass index; SMI: Skeletal muscle mass index; PhA: Phase angle.

**Table 2:** Pearson correlations between anthropometric measurements and phase angle in monozygotic and dizygotic twins.

Variable	Body Weight		BMI		SMI		PhA	
	*r*		*r*		*r*		*r*	
Total (*n* = 168)								
Height (cm)	0.387	**	−0.088		−0.0550		−0.082	
Body Weight (kg)			0.879	**	0.590	**	0.151	
BMI (kg/m^2^)					0.655	**	0.189	*
SMI (kg/m^2^)							0.467	**
MZ (*n* = 150)								
Height (cm)	0.370	**	−0.094		−0.066		−0.107	
Body Weight (kg)			0.885	**	0.618	**	0.127	
BMI (kg/m^2^)					0.681	**	0.163	*
SMI (kg/m^2^)							0.390	**
DZ (*n* = 18)								
Height (cm)	0.527	*	−0.060		−0.029		−0.029	
Body Weight (kg)			0.813	**	0.376		0.171	
BMI (kg/m^2^)					0.464		0.232	
SMI (kg/m^2^)							0.605	**

Note: * *p* < 0.05; ** *p* < 0.01. BMI: Body mass index; SMI: Skeletal muscle mass index; PhA: Phase angle; MZ: Monozygotic twins; DZ: Dizygotic twins.

**Table 3:** Univariate ACE model fitting and estimated values for anthropometric measurements and phase angle.

Variable	−2LL	AIC	*p*-Value	A (95% CI)	C (95% CI)	E (95% CI)
Height (cm)						
ACE	1782	1119	-	-	-	-
AE	1783	1117	0.558	0.932 (0.908–0.950)	-	0.068 (0.050–0.091)
CE	1831	1165	<0.01	-	0.888 (0.851–0.916)	0.112 (0.084–0.149)
E	2092	1424	<0.01	-	-	-
Body Weight (kg)						
ACE	2228	1564	-	-	-	-
AE	2228	1562	1.000	0.758 (0.682–0.817)	-	0.242 (0.183–0.318)
CE	2241	1575	<0.01	-	0.704 (0.619–0.772)	0.296 (0.228–0.381)
E	2356	1688	<0.01	-	-	-
BMI (kg/m^2^)						
ACE	1568	904.0	-	-	-	-
AE	1568	902.0	1.000	0.718 (0.630–0.786)	-	0.282 (0.214–0.370)
CE	1585	919.1	<0.01	-	0.645 (0.548–0.725)	0.355 (0.275–0.451)
E	1675	1007	<0.01	-	-	-
SMI (kg/m^2^)						
ACE	699.8	55.83	-	-	-	-
AE	700.4	54.43	0.439	0.513 (0.457–0.625)	-	0.487 (0.375–0.543)
CE	700.9	54.89	0.304	-	0.467 (0.337–0.579)	0.533 (0.421–0.663)
E	740.1	92.10	<0.01	-	-	-
PhA (°)						
ACE	1783	1119	-	-	-	-
AE	1783	1117	0.558	0.506 (0.341–0.642)	-	0.494 (0.358–0.659)
CE	1831	1165	<0.01	-	0.281 (0.137–0.414)	0.718 (0.585–0.863)
E	2092	1424	<0.01	-	-	-

Note: BMI: Body mass index; SMI: Skeletal muscle mass index; PhA: Phase angle; *p*-value for statistical difference versus ACE model. −2LL: −2 log-likelihood; AIC: Akaike’s information criterion; A: Additive genetic factor; C: Shared environmental factor; E, non-shared environmental factor; CI: Confidence interval.

## Supplementary Materials

The following are available online at https://www.mdpi.com/1660-4601/17/21/7810/s1, Table S1: Univariate ADE model fitting and standardized path coefficients for anthropometric measurements and phase angle.
