# Supplementary material for: Heritability and Environmental Correlation of Phase Angle with Anthropometric Measurements: A Twin Study
Source: Int J Environ Res Public Health. 2020 Oct 26;17(21):7810. doi: 10.3390/ijerph17217810 (PMC7662672; doi:10.3390/ijerph17217810)
Supplement: Supplementary file 1 [file ijerph-17-07810-s001.pdf]

International Journal of
Environmental Research and Public Health

Table S1. Univariate ADE model fitting and estimated values for anthropometric measurements and phase angle.

Variable	−2LL	AIC	*p*-value	A (95% CI)	D (95% CI)	E (95% CI)
Height (cm)						
ADE	893.0	565.0	-	-	-	-
AE	893.0	563.0	1.000	0.932 (0.909–0.950)	-	0.068 (0.050–0.091)
E	1046	714.1	<0.001	-	-	-
Body Weight (kg)						
ADE	1113	784.7	-	-	-	-
AE	1113	782.9	0.685	0.758 (0.682–0.817)	-	0.242 (0.183–0.318)
E	1178	845.9	<0.001	-	-	-
BMI (kg/m^2)						
ADE	782.2	454.2	-	-	-	-
AE	783.4	453.4	0.278	0.718(0.630–0.786)	-	0.282(0.214–0.370)
E	837.5	505.5	<0.001	-	-	-
SMI (kg/m^2)						
ADE	349.8	31.75	-	-	-	-
AE	349.8	29.75	1.000	0.512 (0.457–0.625)	-	0.487 (0.375–0.543)
E	369.8	47.81	<0.001	-	-	-
PhA (°)						
ADE	367.2	39.23	-	-	-	-
AE	369.3	39.26	0.154	0.506 (0.341–0.642)	-	0.494 (0.358–0.659)
E	383.6	51.63	<0.001	-	-	-

Note: BMI: Body mass index; SMI: Skeletal muscle mass index; PhA: Phase angle; *p*-value for statistical difference versus ACE model. −2LL: −2 log-likelihood; AIC: Akaike's information criterion; A: additive genetic factor; D: dominance factor; E, non-shared environmental factor; CI: confidence interval.
